# Coordinated Contribution of NADPH Oxidase- and Mitochondria-Derived Reactive Oxygen Species in Metabolic Syndrome and Its Implication in Renal Dysfunction

**DOI:** 10.3389/fphar.2021.670076

**Published:** 2021-05-04

**Authors:** Hewang Lee, Pedro A Jose

**Affiliations:** ^1^ Department of Medicine, The George Washington University School of Medicine and Health Sciences, Washington, DC, United States; ^2^ Department of Pharmacology and Physiology, The George Washington University School of Medicine and Health Sciences, Washington, DC, United States

**Keywords:** metabolic syndrome, mitochondria, nicotinamide-adenine dinucleotide phosphate oxidase, NLRP3, oxidative stress, reactive oxygen species, pyruvate dehydrogenase complex, renal dysfunction

## Abstract

Metabolic syndrome (MetS), a complex of interrelated risk factors for cardiovascular disease and diabetes, is comprised of central obesity (increased waist circumference), hyperglycemia, dyslipidemia (high triglyceride blood levels, low high-density lipoprotein blood levels), and increased blood pressure. Oxidative stress, caused by the imbalance between pro-oxidant and endogenous antioxidant systems, is the primary pathological basis of MetS. The major sources of reactive oxygen species (ROS) associated with MetS are nicotinamide-adenine dinucleotide phosphate (NADPH) oxidases and mitochondria. In this review, we summarize the current knowledge regarding the generation of ROS from NADPH oxidases and mitochondria, discuss the NADPH oxidase- and mitochondria-derived ROS signaling and pathophysiological effects, and the interplay between these two major sources of ROS, which leads to chronic inflammation, adipocyte proliferation, insulin resistance, and other metabolic abnormalities. The mechanisms linking MetS and chronic kidney disease are not well known. The role of NADPH oxidases and mitochondria in renal injury in the setting of MetS, particularly the influence of the pyruvate dehydrogenase complex in oxidative stress, inflammation, and subsequent renal injury, is highlighted. Understanding the molecular mechanism(s) underlying MetS may lead to novel therapeutic approaches by targeting the pyruvate dehydrogenase complex in MetS and prevent its sequelae of chronic cardiovascular and renal diseases.

## Introduction

Metabolic syndrome (MetS), a complex of interrelated risk factors for cardiovascular disease and diabetes, is comprised of central obesity (increased waist circumference), hyperglycemia, dyslipidemia (high triglyceride blood levels, low high-density lipoprotein blood levels), and increased blood pressure (Alberti et al., 2009; Nilsson et al., 2019). The presence of three of the five criteria qualifies a diagnosis of MetS: waist circumference ≥102 cm for males and ≥88 cm for females (≥90 cm and ≥80 cm for Asians, respectively); fasting blood glucose ≥5.6 mmol/L or drug treatment for elevated blood glucose; blood triglycerides ≥1.7 mmol/L or drug treatment for elevated blood triglycerides; reduced blood high-density lipoprotein cholesterol (<1.0 mmol/L for females and <1.4 mmol/L for males) or drug treatment for reduced blood high-density lipoprotein cholesterol; and systolic blood pressure ≥130 mmHg and/or diastolic blood pressure ≥85 mmHg or antihypertensive drug treatment in a patient with a history of hypertension (Alberti et al., 2009, Nillson et al., 2019). MetS is characterized by systemic oxidative stress, chronic inflammation, and insulin resistance (Furukawa et al., 2004; Grundy, 2016).

Oxidative stress, the principal pathological basis for MetS (Spahis et al., 2017), is a state of an imbalance of the production and degradation of reactive oxygen and nitrogen species, resulting in an increase in reactive oxygen and nitrogen species (Egea et al., 2017). Reactive oxygen species (ROS) and nitrogen species are reactive derivatives of oxygen metabolism. Excessive ROS damage lipids, proteins, nucleic acids, and carbohydrates, resulting in a chronic increase in the production of proinflammatory cytokines and cellular inflammation (Touyz et al., 2020). Experimental and clinical evidence suggests that oxidative stress increases leptin secretion by adipocytes, induces β-cell dysfunction, and impairs insulin signaling and glucose tolerance, leading to the development of insulin resistance (Newsholme et al., 2019).

Oxidative stress occurs when energy supply exceeds energy expenditure and as a consequence, adipocytes undergo proliferation and hypertrophy, leading to visceral obesity (Jia et al., 2016; Masschelin et al., 2020; Prasun, 2020). There is a direct correlation between the plasma level of malondialdehyde (MDA), a biomarker of lipid peroxidation and total oxidant status, and the body mass index in subjects with MetS (Furukawa et al., 2004; Maslov et al., 2019). Oxidative stress activates a series of stress pathways, modulates transcription factors, increases the generation of adipokines and cytokines, and causes derangements in metabolism, including impaired glucose tolerance, dyslipidemia, ectopic lipid accumulation, gut microbiota dysbiosis, and hypertension, ultimately leading to MetS (Rani et al., 2016; Vona et al., 2019). MetS markedly increases the risk of the occurrence and progression of chronic cardiovascular and renal diseases (Zhang and Lerman, 2017; Senoner and Dichtl, 2019; Podkowińska and Formanowicz, 2020).

Knowledge of the sources of ROS, the amount and type of reactive species produced, the cellular signaling involved, and the affected targets, is critical in understanding the initiation and progression of MetS. In this review, we discuss the generation of ROS from two major sources (NADPH oxidases and mitochondria), downstream signaling pathways, pathophysiological processes, and progression of MetS.

## Reactive Oxygen Species Generation by Nicotinamide-Adenine Dinucleotide Phosphate Oxidases and Mitochondria

ROS can be free radicals and non-free radicals (Zeng et al., 2009; Gutteridge and Halliwell, 2018; Zhang et al., 2019) (Table 1). A free radical contains one unpaired reactive electron in the outer orbit, such as superoxide anion (O_2_-), hydroxyl radical (OH), nitric oxide (NO), carbonate radical anion (CO_3_-), nitrogen dioxide (NO_2_), and alkoxyl/alkyl peroxyl (RO/ROO). A non-free radical does not contain unpaired electron, such as hydrogen peroxide (H_2_O_2_), hypochlorous acid (HOCl), and peroxynitrite (ONOO-) (Zeng et al., 2009).

**TABLE 1:** Characteristics of some major ROS generated by NADPH oxidases and mitochondria^6,7,16–1^ ^8^.

ROS	Free radical	Lifetime	Major targets	Possible candidate as second messenger	Scavengers
Superoxide anion (O_2_^•-^)	Yes	∼50 ms	Fe-S cluster	No	SOD
			NO		
Hydrogen peroxide (H_2_O_2_)	No	∼1 ms	Thiols	Yes	Catalase
			Metals		GPX
					PRDX
					Bilirubin
Hydroxyl radical (OH•)	Yes	∼10^−9^s	Any macromolecule	No	Uric acid
					Vitamin E
Nitric oxide (NO•)	Yes	∼1s	Metals	Yes	NA
Nitrogen dioxide (NO_2_•)	Yes	NA	Thiols	ND	GPX
					PRDX
Hypochlorous acid (HOCl)	No	NA	Amino	No	Uric acid
			Thiols		
Peroxynitrite (ONOO−/ONOOH)	No	∼15°μs	CO2	No	GPX
			Cysteine		PRDX
			Tryptophan		Bilirubin
			Tyrosine		
			Metals		
Carbonate radical anion (CO_3_•-)	Yes	∼10^−6^°s	Thiols	No	ND
			Guanine		
Alkoxyl/Peroxyl radical (RO/ROO•)	Yes	NA	Organic compounds	ND	GPX
					PRDX

Fe-S, iron (II) sulfide; GPX, glutathione peroxidase; PRDX, peroxiredoxin; SOD, superoxide dismutase; NA, not available; ND, not determined.

Superoxide anion (O_2_-), designated as the primary ROS, is derived from O_2_ after receiving an electron from oxidases or the mitochondrial electron transport chain (ETC) (Gutteridge and Halliwell, 2018). O_2_- is then dismutated spontaneously or by superoxide dismutase (SOD) into H_2_O_2_, which is then converted to water by glutathione peroxidase or catalase. O_2_- reacts with nitric oxide (NO) to form ONOO-. Hydroxyl radical (OH) is derived from ONOOH by homolytic fission or from H_2_O_2_ through the Haber-Weiss reaction in the presence of transition metals, such as Fe^2+^, or through HOCl by myeloperoxidase in reaction with O_2_. The formation of lipid radical (L) (not listed in Table 1) is usually initiated by OH^–^, abstracting an allylic hydrogen from unsaturated lipid, which then reacts with O_2_ to form a lipid peroxyl radical (LOO); the abstraction of one H^+^, from an unsaturated lipid, forms lipid hydroperoxide (LOOH) (Zhang et al., 2019). ROS are cleared by antioxidant systems. As aforementioned, O_2_- can be dismutated to H_2_O_2_; H_2_O_2_ can be further decomposed by catalase, glutathione redox cycle, and thioredoxin redox cycle. ROS can be removed by some non-enzymatic compounds, such as vitamins C and E, carotenoids, glutathione, nicotinamide, and flavonoids (Tan et al., 2018).

ROS are generated by specific oxidase enzymes, such as the nicotinamide-adenine dinucleotide phosphate (NADPH) oxidases, xanthine oxidases, arachidonic acid monooxygenases, nitric oxide synthases, cyclooxygenases, cytochrome P450 enzymes, and lipoxygenases, as well as cell organelles, such as mitochondria, peroxisomes, and endoplasmic reticula (Zeng et al., 2009; Jia et al., 2016; Prasun, 2020). The major sources of ROS associated with MetS pathogenesis are NADPH oxidases (DeVallance et al., 2019) and mitochondria (Prasun, 2020).

**FIGURE 1:**
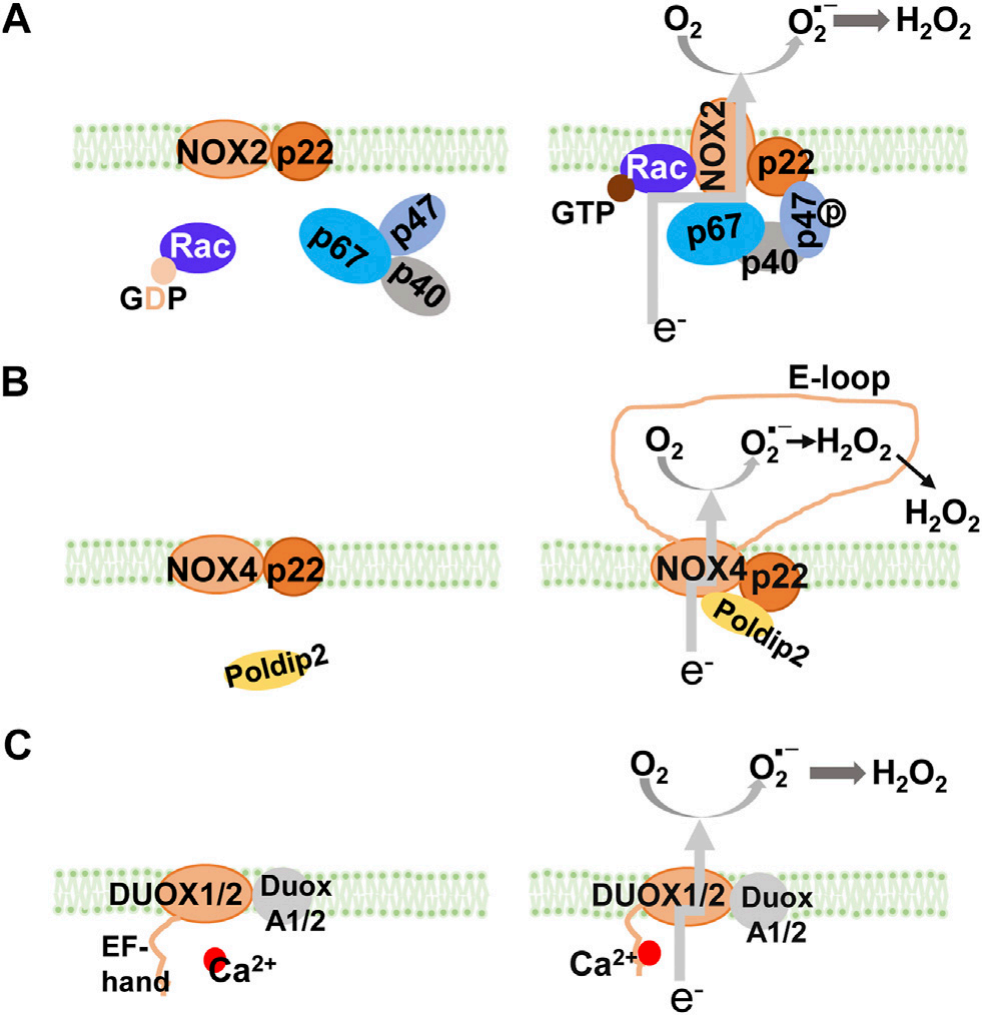
NADPH oxidase isoforms and their assembly. All NOX isoforms have membrane-spanning catalytic subunit that transfers electrons from NADPH through their NADPH and FAD-binding sites and heme groups to reduce O_2_ into superoxide anion **(A)** NOX1, NOX2, and NOX3 are cytosolic activators-dependent with the stabilizing p22^phox^ membrane subunit. Activation of NOX2 requires assembly with cytosolic p47^phox^ (equivalent to NoxO1 in NOX1 and NOX3), p67^phox^ (equivalent to NoxA1 in NOX1 and NOX3), and p40^phox^. In resting cells **(left panel)**, NOX2 and p22^phox^ are associated, co-stabilizing each other. Upon activation **(right panel)**, GDP is exchanged for GTP on Rac. The phosphorylation of p47^phox^ subunit leads to a conformational change, which brings with p67^phox^ and p40^phox^, to form the active NOX2 enzyme complex (NOX1 and NOX3 are not shown) **(B)** NOX4 is constitutively active with the stabilizing p22^phox^ membrane subunit. Its activity can be modulated by Poldip2. Inside the E-loop (3^rd^ extracellular loop), superoxide is rapidly dismutated into H_2_O_2_ **(C)** NOX5, DUOX1, and DUOX2 are Ca^2+^ dependent with EF-hand domains. DUOX1 and DUOX2 (as well as NOX5, not shown) have Ca^2+^ binding domains that undergo conformational change in response to an increase in intracellular Ca^2+^. It is speculated that activation of DUOX1, DUOX2, and NOX5, is through intramolecular protein-protein interaction between the Ca^2+^-binding domain and the C-terminal catalytic domain. GDP, guanosine diphosphate; GTP, guanosine-5′-triphosphate; NADPH, nicotinamide adenine dinucleotide phosphate; NOX, NADPH oxidase.

NADPH oxidase, a major source of O_2_- and H_2_O_2_ (Bedard and Krause, 2007), is comprised of seven structurally similar members, NOX1 to NOX5, and Duox1 and Duox2 (Figure 1). All NADPH oxidase members have two heme groups, six transmembrane domains, and flavin adenine dinucleotide (FAD) and NADPH binding sites (Groemping and Rittinger, 2005). The cytosolic FAD domain receives two electrons from NADPH that are sequentially transferred along the two heme domains which reduce O_2_ to O_2_- (DeVallance et al., 2019). Activation of NOX1, NOX2, and NOX3 requires the assembly of membrane and cytosolic subunits, similar to the classical NADPH oxidase, NOX2 (membrane subunit, p22^phox^, and cytosolic subunits, p47^phox^, p67^phox^, and p40^phox^) to produce O_2_- (Nauseef, 2014). Activation of NOX4 (heterodimerized with p22^phox^) which does not require classical cytosolic subunits or their homologs, produces mainly H_2_O_2_ (Lyle et al., 2009). Activation of NOX5 and Duoxs 1 and 2 does not require cytosolic subunits either, but rather by the binding of calcium to their intracellular EF-hand motifs, resulting in the production of O_2_- and/or H_2_O_2_ (Banfi et al., 2004).

The mitochondrion, the other major source of ROS, in addition to NADPH oxidases, within the cell (Murphy, 2009), continuously metabolizes oxygen which generates ROS (Bhatti et al., 2017). During the process of oxidative phosphorylation, electrons leak from the ETC to oxygen and produce ROS: single electron leak generates O_2_- while two-electron leak generates H_2_O_2_ (Brand, 2016). In general, increasing electron flux through the ETC decreases the probability of unpaired electron leakage to form ROS (Hoffman and Brookes, 2009); ROS production is increased at high ETC rates when ROS scavenging pathways are overwhelmed (Sharaf et al., 2017). However, ROS are also produced during low ETC activity when electron flux is inhibited (Murphy, 2009), because the electron spends more time at reductive centers, increasing the chances of its transfer to oxygen (Gnaiger et al., 1998). The increase in ROS production in both low and high ETC fluxes is evidence of the complicated nature of ROS production in mitochondria under different conditions (Murphy, 2009; Berry et al., 2018). ETC Complex I is believed to be a major source of mitochondrial ROS (Murphy, 2009). In ETC Complex I, electrons enter the NADH/NAD^+^ pool through NAD-linked dehydrogenases, and ROS can be produced from multiple sites (Brand, 2016), when the NADH/NAD^+^ ratio is high in cells (Kussmaul and Hirst, 2006), particularly at the site of dihydrolipoamide dehydrogenase subunit (Bazil et al., 2014). Electrons from NADH flow into the flavin-containing sites to the ETC Complex I quinone-binding site and then to the next isopotential pool ETC Complex II (Brand, 2016). ROS are also produced by the flavin in ETC Complex I, under the condition of reverse electron transport (Chouchani et al., 2014), when the protonmotive force (pmf, including Δψm and ΔpH) is increased (Scialò et al., 2017). In ETC Complex III, electrons are transferred from QH2 to the outer Q-binding site of ETC Complex III (site III Qo), then through cytochrome b566 to ETC Complex III Qi in the Q-cycle, and subsequently down through cytochrome C, and ETC Complex IV to oxygen (Brand, 2016). ROS production is dependent on kinetics and redox status during the Q cycle (Dröse and Brandt, 2012); high ΔpH component of the pmf leads to an increase in ROS production from ETC Complex III (Wong et al., 2017). When the electrons are transferred from ETC Complexes I, III, and IV, the isopotential drops, the protons are pumped across the inner mitochondrial membrane, and energy is conserved as the pmf, which is driven for ATP synthesis (Brand, 2016). In addition to the ETCs, ROS in the mitochondria can also be produced by enzymes, such as pyruvate dehydrogenases which transfer electrons to oxygen (Quinlan et al., 2014; Ambrus et al., 2015).

The concentration of ROS within cells is the net balance of ROS producing and antioxidant systems. Endogenous antioxidants consist of enzymatic and non-enzymatic scavengers (Ahmadinejad et al., 2017). Enzymatic scavengers include superoxide dismutases (SODs), catalases, and various peroxidases, and non-enzymatic antioxidants include vitamin C and E, carotenoids, glutathione, lipoic acid and transition-metal ions (Moldogazieva et al., 2018). The three isoforms of SODs in mammals, SOD1 in the cytoplasm and the mitochondrial intermembrane space, SOD2 in the mitochondrial matrix, and SOD3 in the extracellular space, scavenge superoxide into H_2_O_2_ and inhibit the formation of ONOO^–^ from NO (Ahmadinejad et al., 2017). Catalases and peroxidases catalyze the conversion of H_2_O_2_ into water (Moldogazieva et al., 2018). However, it is more dependent on rates of ROS production than on the rates of antioxidant scavenging (Munro and Treberg, 2017). For simplicity, this review does not discuss other cellular producers of ROS and enzymatic or non-enzymatic antioxidants.

## Crosstalk Between Nicotinamide-Adenine Dinucleotide Phosphate Oxidases and Mitochondria-Derived Reactive Oxygen Species

ROS-induced ROS release is a self-amplified phenomenon initially observed in mitochondrial ROS (Mito-ROS) generation triggered by ROS released from dysfunctional mitochondria (Zorov et al., 2006). This positive feedback cycle is also observed later in the ROS generation between the activation of NADPH oxidases and mitochondria, which becomes a vicious cycle (Daiber, 2010; Zinkevich and Gutterman, 2011). The crosstalk between the two major sources of ROS augments oxidative stress (Daiber, 2010; Dikalov, 2011). ROS derived from NADPH oxidases promote Mito-ROS production and vice versa, such that Mito-ROS activate NADPH oxidases to generate more ROS (Daiber et al., 2017; Egea et al., 2017; Dikalov and Dikalova, 2019).

The phenomenon that ROS derived from NADPH oxidases promote Mito-ROS production is well described in the activation of NADPH oxidases by the renin-angiotensin (Ang II)-aldosterone system (RAAS) (Kimura et al., 2005). Activation of NADPH oxidases located in both the plasma and cytosolic membranes generates O_2_-. Its dismutation into H_2_O_2_ activates PKC-ε and induces phosphorylation-dependent mitochondrial K_ATP_ channel (Mito-K_ATP_) opening (Costa and Garlid, 2008a). This increases K^+^ influx, decreases mitochondrial membrane potential (ΔΨm), and induces mitochondrial O_2_- production by reverse electron transfer, which is released by the opening of mitochondrial permeability transition pore (Dikalov and Dikalova, 2019). The matrix alkalinization following K^+^ influx due to Mito-K_ATP_ opening results in an increase in H_2_O_2_ formation (Daiber et al., 2020). The O_2_- production can be blocked by the specific Mito-K_ATP_ inhibitor 5-hydroxydecanoate (Daiber et al., 2020). In preadipocytes and adipocytes (Baret et al., 2017), endothelial cells (Coughlan et al., 2009), and renal mesangial cells (Xie et al., 2012), activation of NADPH oxidases by advanced glycation end-products (AGEs) enhances mitochondrial biogenesis, decreases mitochondrial electron transport, and overproduction of Mito-ROS. The increase in the production of Mito-ROS is attenuated by knockdown of NADPH oxidase subunits or NOX inhibitors (Ahmed et al., 2012; Daiber et al., 2017; Dikalov and Dikalova, 2019), supporting the existence of ROS crosstalk from NADPH oxidases to the mitochondria.

Mito-ROS can also activate NADPH oxidases to produce ROS. O_2_- generated in the mitochondria is mainly dismutated into H_2_O_2_. Mito-ROS (mainly O_2_- and H_2_O_2_) are released to the cytosol via mitochondrial permeability transition pore (mPTP), inner membrane anion channel (IMAC), voltage-dependent anion channel, and aquaporins, or via diffusion due to increased mitochondrial permeability under proinflammatory conditions (Daiber, 2010; Dikalov, 2011; Brand, 2016; Daiber et al., 2017). The cytosolic H_2_O_2_ derived from the mitochondria activates redox-sensitive tyrosine kinases (c-SRCs) and protein kinases (PKCs), which subsequently phosphorylate NADPH oxidases, facilitating the assembly of NADPH oxidase subunits in the membranes, which amplifies the ROS production (Dikalov, 2011; Kröller-Schön et al., 2014; Zhang et al., 2014; Daiber et al., 2017; Daiber et al., 2020). Inhibition of Mito-ROS by Mito-TEMPO effectively suppresses the activities of NADPH oxidases and ROS production (Park et al., 2015; Ni et al., 2016). Depletion of mitochondrial SOD2 increases both basal and Ang II-stimulated NADPH oxidase activity, whereas overexpression of SOD2 attenuates ROS production from NADPH oxidases by scavenging mito-ROS (Dikalov, 2011; Egea et al., 2017). The regulation of NADPH oxidases by Mito-ROS is observed *in vivo,* as well (Tran et al., 2017). All of these studies show the existence of ROS crosstalk from mitochondria to NADPH oxidases.

**FIGURE 2:**
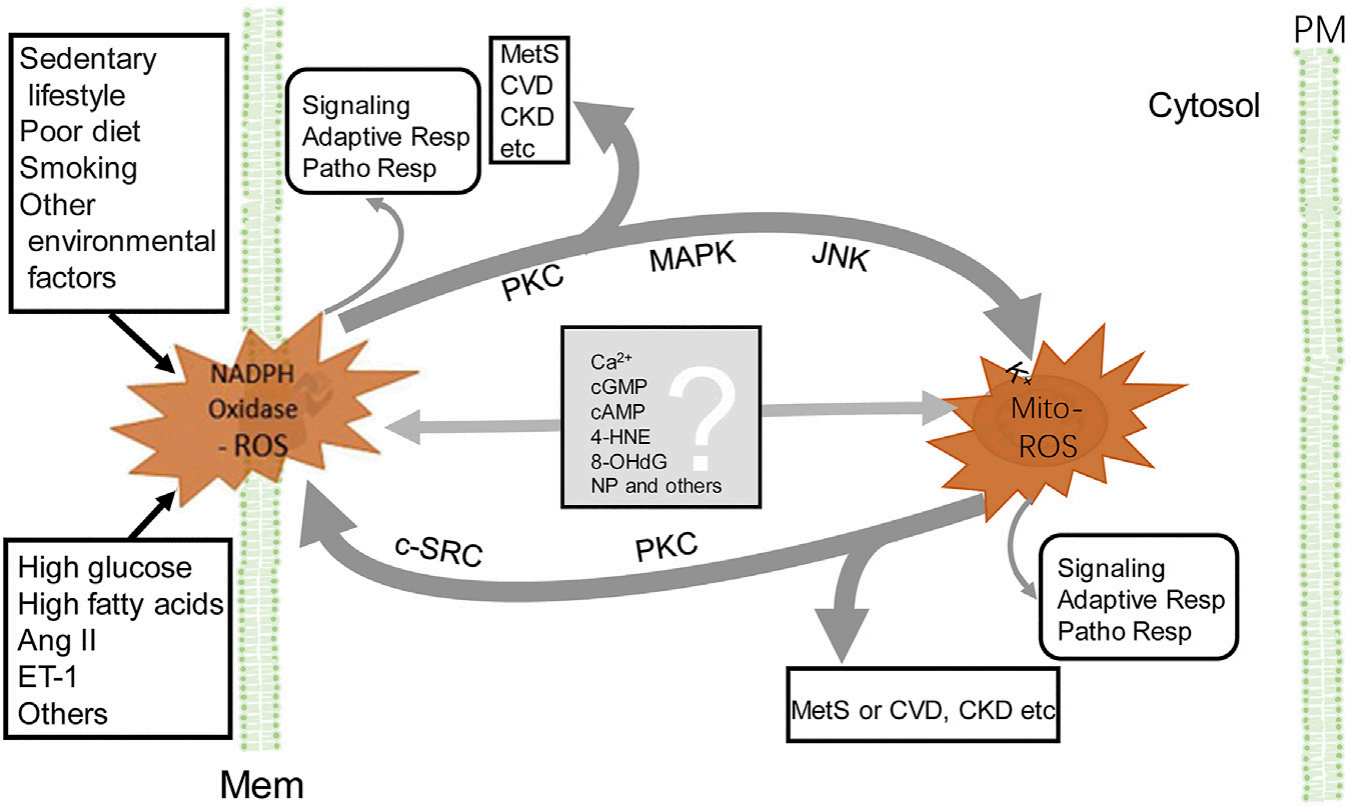
Crosstalk between NADPH oxidase and mitochondria-derived ROS. NADPH oxidase activation, by endogenous and exogenous stimulation (for example Ang II), produces ROS, which stimulate PKC, MAPK, and JNK, leading to the increase in Mito-ROS production (see the text). ROS, released from mitochondria, activate redox-sensitive PKC and c-SRC, which phosphorylate NADPH oxidases and increase ROS production by NADPH oxidases. The Mito-ROS trigger NADPH oxidases activation and generation of ROS and vice versa, resulting in a vicious circle (thick gray lines). The converged increase in ROS contributes to the pathogenesis of MetS, CVD, and CKD, among others. In addition to PKC, other potential molecules (listed in the gray box) relay the signaling between NADPH oxidase and mitochondria. ROS generated by either NADPH oxidase or mitochondria exert their individual actions, such as signaling, adaptive responses, and pathophysiological responses (very thin gray line arrows). 4-HNE, 4-hydroxynonenal; 8-OHdG, 8-hydroxy-20-deoxyguanosine; Adaptive Resp, adaptive responses; CKD, chronic kidney disease; c-SRC, proto-oncogene tyrosine-protein kinase; CVD, cardiovascular disease; MAPK, mitogen-activated protein kinase; PM, plasma membrane and other cellular membranes (Mem), such as in endoplasmic reticula, endosomes etc; MetS, metabolic syndrome; Mito-ROS, ROS generated from mitochondria (right side); NADPH Oxidase-ROS, ROS generated from NADPH oxidases (left side); NP, nanoparticles or microparticles; Patho Resp, pathological responses; PKC, protein kinase C. For simplicity, other ROS-generating sources and enzymatic and non-enzymatic antioxidants are not shown.

The crosstalk between NADPH oxidase and mitochondrial ROS provides a network of intracellular redox regulation (Figure 2). Each ROS source has so-called “redox switches” that confer activation upon oxidation (Egea et al., 2017). The on/off of the redox switches results in activation or inactivation of effector proteins and transcription factors that function in a wide array of cellular physiological and pathophysiological responses (Daiber et al., 2017). For example, in endothelial cells, ROS activate PKC and protein tyrosine kinase 2-dependent phosphorylation and uncoupling of endothelial NO synthase, desensitization of soluble guanylate cyclase, nitration of prostacyclin and increase in cyclooxygenase activity, and augmentation of vasoconstriction and resulting hypertension induced by endothelin-1 (Li et al., 2003; Chen et al., 2012; Wu et al., 2014; Daiber et al., 2017). The crosstalk produces increased levels of ROS (Egea et al., 2017), resulting in a vicious cycle (Daiber, 2010; Dikalov, 2011; Daiber et al., 2020). Because ROS lifetime is short (Table 1), there must be some mediators to carry out the crosstalk from NADPH oxidases to mitochondria and vice versa (Figure 2). It is speculated that calcium (Görlach et al., 2015), cGMP (Costa et al., 2008b), cAMP (Palmeira et al., 2019), 4-hydroxy-2-nonenal (Xiao L. et al., 2017), 8-hydroxy-20-deoxyguanosine (Termini, 2000), and microparticles (Uusitalo and Hempel, 2012), among others, are the candidates that relay the signal from one to another. This synergistic regulation may not necessarily represent a general mechanism, depending on the highly dynamic spatiotemporal relationship between these two major ROS sources. The mitochondrion, itself, is a very dynamic organelle, which can be physically associated with NADPH oxidases through the contact sites between the mitochondria and ER, endosomes, or the plasma membrane. The NADPH oxidase isoform, NOX4, which directly produces H_2_O_2_, is expressed in the mitochondria (Hirschhäuser et al., 2015). As aforementioned, the crosstalk between mitochondria- and NADPH oxidase-generated ROS can result in the vicious cycle of ROS formation, resulting in oxidative stress, which contributes to the development and progression of pathological conditions, including MetS (Figure 2).

## Nicotinamide-Adenine Dinucleotide Phosphate Oxidase-Reactive Oxygen Species Signaling and Pathophysiological Effects in Metabolic Syndrome

In response to extracellular and intracellular metabolic alterations or damage, ROS, generated by NADPH oxidases located in plasma or cytosolic membranes, trigger a series of physiological, adaptive, and subsequently pathological responses (Yang et al., 2021). These regulate transcriptional factors and gene expression, resulting in metabolic reprogramming, that results in the initiation and progression of MetS (Perez-Martinez et al., 2012; Purkayastha and Cai, 2013). NADPH oxidases are widely expressed in various cells: NOX2 in macrophage and neutrophils; NOX2, NOX4, with some NOX1 and NOX5 in endothelial cells (DeVallance et al., 2019); NOX4 and NOX1 in vascular smooth muscle cells (VSMCs) (Akoumianakis and Antoniades, 2019); NOX4 in adipocytes (DeVallance et al., 2019); and NOX2, NOX4, and NOX5 in renal epithelial cells (Yang et al., 2020; Yang et al., 2021), all playing critical roles in the pathogenesis of MetS.

**FIGURE 3:**
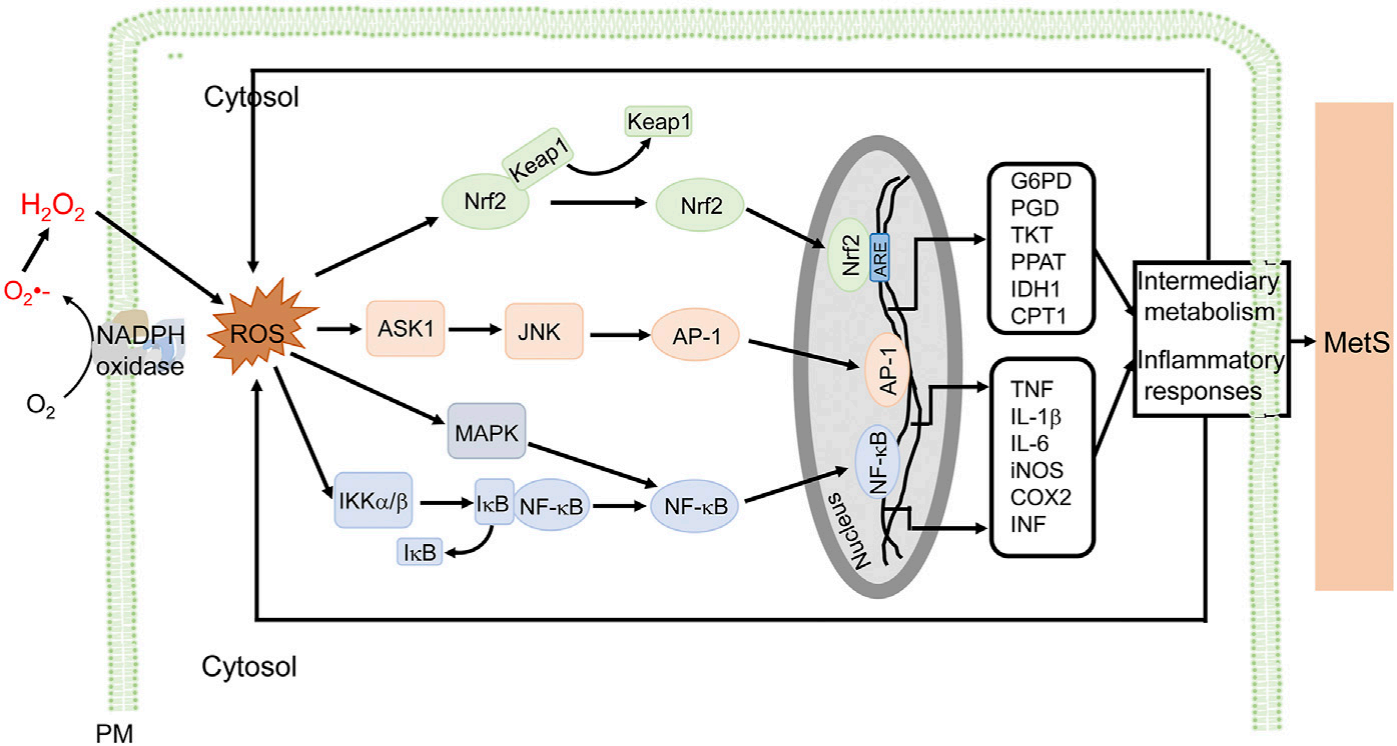
NADPH oxidase-derived ROS signaling and cellular effects in MetS. ROS derived from NADPH oxidases are involved in the inflammatory responses associated with MetS, in macrophages, for example. ROS stimulate NF-κB, via MAPK and IKKα/β, and AP-1, via ASK1 and JNK, which promote the transcription of proinflammatory genes (e.g., TNF, IL-1β, IL-6, iNOS, COX2, and INF), resulting in inflammatory responses associated with MetS. ROS also activate Nrf2 to induce genes that regulate intermediary metabolism (e.g., G6PD, PGD, TKT, PPAT, IDH1, and CPT1) (only some genes are listed). The antioxidant effect and modulation of metabolism are adaptive responses to ROS-induced proinflammatory responses and altered metabolism. ROS exert a wide range of effects such as inflammation, insulin resistance, adipocyte proliferation, altered metabolism, among others in diverse cells, including macrophages, endothelial cells, vascular cells, adipocytes, preadipocytes, and epithelial cells. The ROS-mediated signaling and cellular efforts are cell type-specific that are overlapping. For simplicity and clarity, only part of signaling and cellular effects in macrophages are illustrated. AP-1, activator protein 1; ASK1, apoptosis signal-regulating kinase 1; COX2, cyclooxygenase-2; CPT1, carnitine palmitoyltransferase 1; G6PD, glucose-6-phosphate dehydrogenase; IDH1, isocitrate dehydrogenase 1; IL, interleukin; iNOS, inducible nitric oxide synthase; JNK, c-Jun N-terminal kinases; Keap1, Kelch-like ECH-associated protein 1; IKK, IκB kinase; MAPK, mitogen-activated protein kinase; MetS, metabolic syndrome; PM, plasma membrane and other cellular membranes; NF-κB, nuclear factor kappa-light-chain-enhancer of activated B cells; Nrf2, nuclear factor-erythroid 2 p45-related factor 2; PGD, 6-phosphogluconate dehydrogenase; PPAT, phosphoribosyl pyrophosphate aminotransferase; TKT, transketolase.

ROS derived from NADPH oxidases are important in the inflammation associated with MetS (Figure 3). In immune cells, ROS (mainly O_2_-) activate the MAP kinase signaling pathway and induce the translocation of nuclear factor kappa B (NF-κB) from the cytosol to the nucleus, where it promotes the synthesis of tumor necrosis factor (TNF), interleukin-6 (IL-6), and inducible nitric oxide synthase (iNOS), leading to proinflammatory responses (Maslov et al., 2019; Touyz et al., 2020; Verzola et al., 2020). In endothelial cells, the increase in NOX activity activates NK-κB and activator protein-1 (AP-1) transcription factors, and upregulates P-selectin and fractalkine, causing the adhesion of monocytes to the endothelium (Manduteanu et al., 2010). In addition, ROS promote AGEs formation, activate c-Jun N-terminal kinases (JNKs), induce expression of heat shock factor 1(HSF1), plasminogen activator inhibitor-1 (PAI-1), and monocyte chemoattractant protein-1 (MCP-1), increase vascular permeability, and recruit immune cells into the sites of inflammation (Sangle et al., 2010). The atypical PKCζ activates NOX2 through phosphoinositide 3-kinase (PI3K)γ by TNF in endothelial cells (Frey et al., 2006). In adipocytes, H_2_O_2_ generated mainly by NOX4, promotes the activation of anti-apoptotic kinase (Akt), Janus kinases (JAKs), and extracellular signal-regulated kinase (ERK1/2), followed simultaneously by the activation of transcription factor, signal transducer and activator of transcription (STAT), resulting in an increase in MCP-1, TNF, and IL-6 production (Maslov et al., 2019). In VSMCs, ROS activate NF-κB, AP-1 and induce gene expression responsible for migration and proliferation of VSMCs (Akoumianakis and Antoniades, 2019). In human aortic VSMCs, transcription factors AP-1 and STAT1/STAT3 interact with the NOX5 promoter; depletion or inhibition of NF-B, AP-1, or STAT1/3 reduces the interferon-induced Ca^2+^-dependent NOX activation and NOX5 expression (Manea et al., 2012). In addition to the increase in the activity of NOX and associated mediators, the aforementioned transcription factors increase NOX activity and ROS production, contributing to the “vicious cycle” in the development of MetS (Manea et al., 2015). NADPH oxidase inhibitors reduce proinflammatory cytokines and decrease hyperlipidemia (Furukawa et al., 2004) in high-glucose diet-fed mice which are hyperglycemic and have oxidative stress and inflammation (McCracken et al., 2018). High-fat diet in mice overexpressing p22^phox^ induces an inflammatory state and MetS phenotype (Youn et al., 2014). Plasma levels of proinflammatory cytokines and adipocytokines are higher in MetS patients than normal subjects (Kim et al., 2016).

Oxidative stress can trigger obesity. NADPH oxidase overexpression, particularly NOX4, increases ROS production, promotes preadipocyte differentiation into adipocytes (Schröder et al., 2009), and induces adipocyte proliferation (Macleod, 2008) and differentiation from adipose-derived stem cells (Kanda et al., 2011). ROS, by stimulation of protein phosphatase 2A and inhibition of cyclin-dependent kinases (Magenta et al., 2008; Burhans and Heintz, 2009), induce dephosphorylation of retinoblastoma protein and activate the transcription factor E2F, a critical regulator of cell proliferation genes. E2F accelerates the re-entry of preadipocytes and adipocytes into the cell cycle (Macleod, 2008). In addition, ROS activation of cyclins D and E allows resting cells to enter into the cell cycle; c-Myc, through ERK, activates cyclin A which promotes proliferation of adipocytes and VSMCs (Macleod, 2008). Subsequently, the expression of p21 and p27, which are cyclin-dependent kinase inhibitors, is increased, facilitating adipocyte differentiation (Macleod, 2008; Rani et al., 2016). Furthermore, in mouse embryonic fibroblasts and adipocytes, ROS also activate C/enhancer-binding protein β and increase peroxisome proliferator-activated receptor (PPAR) γ, promoting adipogenesis and lipogenesis (Shin et al., 2020).

NADPH oxidases are critical in the development of insulin resistance and abnormal glucose and lipid metabolism associated with MetS. NOX4 is required for the physiological actions of insulin. ROS oxidize cysteine residues of protein tyrosine phosphatase (PTP)1B, facilitate the tyrosine phosphorylation of insulin receptor substrate (IRS)1, and glucose uptake (Guichard et al., 2008). However, excessive ROS interfere with insulin signaling, increasing JNK1-mediated IRS1 phosphorylation and proteasomal degradation, impairing insulin-stimulated IRS1 redistribution and PI3K activity, and reducing Akt/PKB phosphorylation (Guichard et al., 2008). JNK1 and MAP kinases activate AP-1, which increases the transcription of inflammatory genes and eventually produces insulin resistance (Bedi et al., 2019). Oxidative stress in pancreatic islets induces β-cell apoptosis through the Bcl-2/Bax pathway (Liang et al., 2017). β-cell dysfunction decreases insulin secretion, resulting in hyperglycemia and more hyperlipidemia (Guichard et al., 2008). In skeletal muscle cells, pigment epithelium-derived factor, a multifunctional serpin implicated in insulin resistance, induces NADPH oxidase-dependent ROS production, enhances phospholipase A2 activity, resulting in lipolysis to produce free fatty acids and stimulation of glycolysis (Carnagarin et al., 2016).

ROS, through vasoactive and renal sodium transport inhibitors or enhancers, such as dopamine, angiotensin II, endothelin 1, and urotensin I, regulate blood pressure by actions in the vasculature and kidney, among others (Touyz et al., 2020; Yang N et al., 2020; Yang S et al., 2021). In the kidney, excessive ROS production increases afferent arteriolar tone and tubular sodium reabsorption and impairs tubuloglomerular feedback (Yang et al., 2020), all of which are involved in the regulation of blood pressure. The overall effect of ROS on renal sodium transport is nephron-segment specific and time- and concentration-dependent (Cuevas et al., 2013; Gonzales-Vicente et al., 2019; Yang et al., 2020). In the renal proximal tubule, NOX2, NOX4, and NOX5 play important roles in the regulation of sodium transport; an increase in ROS production increases sodium transport and subsequently, blood pressure (Han et al., 2008; Li et al., 2009; Yu et al., 2014; Yang et al., 2021). In the vasculature, ROS activate ERK1/2, p38MAPK, and JNK, promoting VSMC proliferation, migration, and inflammation. ROS also activate tyrosine kinases, including c-Src, PI3K/Akt, FAK, and receptor tyrosine kinases, stimulating NFκB activity, STAT1, AP-1, and hypoxia-inducible factor 1 (HIF-1]), leading to an increase in the expression of proinflammatory genes, production of chemokines and cytokines, and recruitment and activation of immune cells that promote vascular inflammation, proliferation, and contraction. The endothelial dysfunction and vascular remodeling result in hypertension and vascular damage (DeVallance et al., 2019; Touyz et al., 2019; Guerby et al., 2021).

Taken together, NADPH oxidases in macrophages and other immune cells, adipocytes, endothelial cells, VSMCs, and renal epithelial cells play important roles in the initiation and progression of MetS by inducing inflammatory responses, adipogenesis and lipogenesis, insulin resistance, metabolic derangements, and increase in renal sodium transport and blood pressure. However, the activation of the transcriptional factor, nuclear factor-erythroid 2 p45-related factor 2 (Nrf2), may also combat the inflammation and altered metabolism (Hayes and Dinkova-Kostova, 2014; He et al., 2020). Activation of Nrf2 upregulates antioxidant and detoxification genes to repair and degrade the damaged macromolecules (Hayes and Dinkova-Kostova, 2014; Saha et al., 2020). Nrf2 also facilitates NADPH regeneration through pentose phosphate pathway and increases β-oxidation of fatty acids (Hayes and Dinkova-Kostova, 2014; Dinkova-Kostova and Abramov, 2015). It is speculated that if adaptive responses cannot resolve the damage caused by inflammation, insulin resistance, and metabolic abnormalities, the pathological processes eventually result in MetS.

## Mitochondria-Derived Reactive Oxygen Species Signaling and Pathophysiological Effects in Metabolic Syndrome

As an inevitable byproduct, mitochondrial ROS must be maintained in a certain range for normal mitochondrial metabolism that involves mPTP and IMAC (Brady et al., 2006). Reversible opening of mPTP and IMAC apparently responds to the inter- and intra-mitochondrial ROS level to release ROS from the mitochondria. This process can play a physiological role to remove unwanted or damaged proteins, organelles (including mitochondria) and cells or cause pathological effects that damage essential proteins, lipids, and nucleic acids, and even eliminate vital and functional mitochondria and cells (Zorov et al., 2014). Therefore, mito-ROS can serve as one of damage-associated molecular patterns (DAMPs). NLRP3 inflammasomes can sense the DAMP-associated danger signals (Schroder and Tschopp, 2010; Abderrazak et al., 2015) and contribute to metabolic reprogramming that leads to the initiation and progression of MetS (Mastrocola et al., 2018).

**FIGURE 4:**
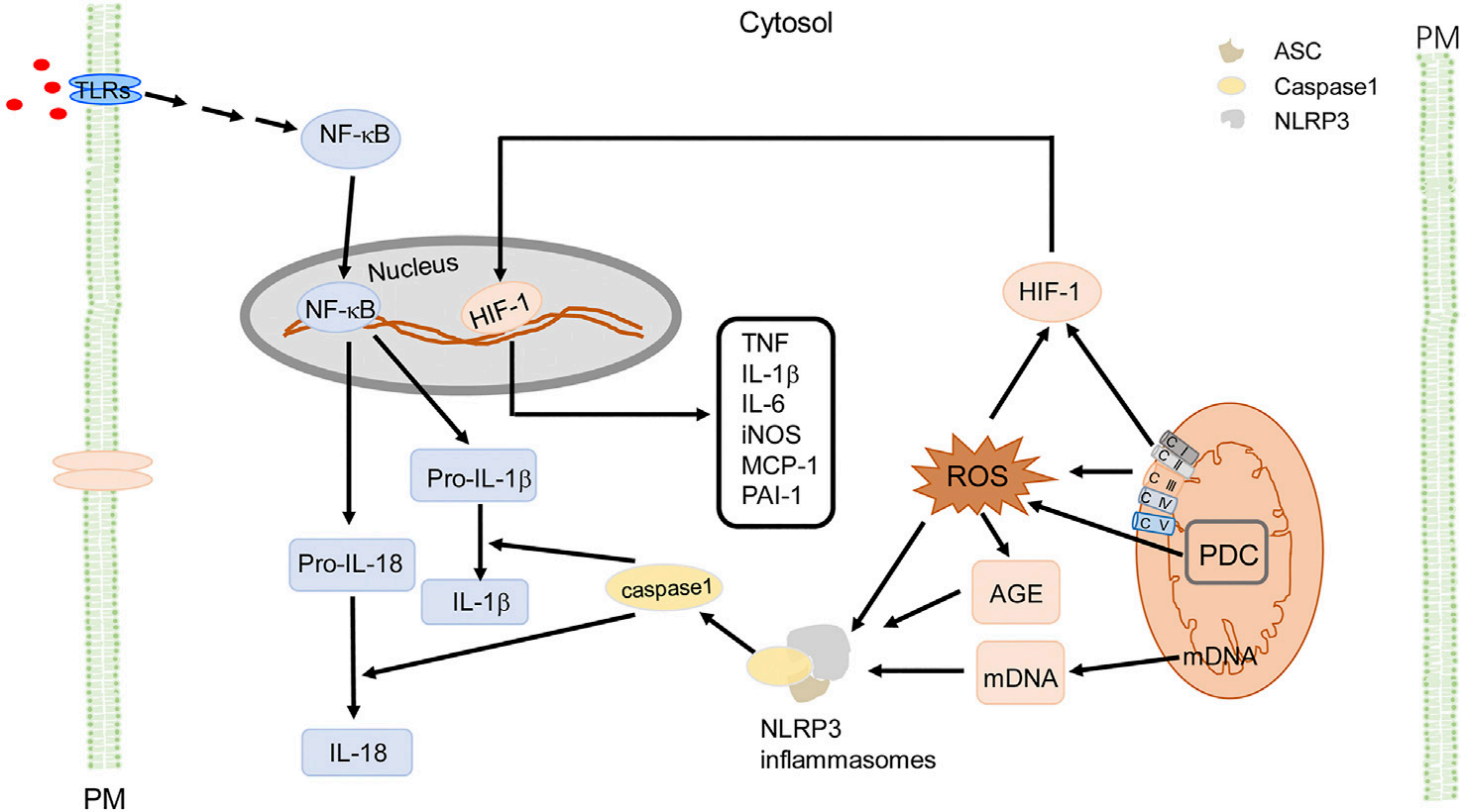
Mito-ROS signaling and cellular effects in MetS. Mito-ROS, produced mainly by ETC (mainly C I and C III) during oxidative phosphorylation and enzymes like pyruvate dehydrogenase complex (PDC), provide the signaling to trigger NLRP3 inflammasome oligomerization with adaptor protein ASC and pro-caspase-1. NLRP3 activation can be primed by TLRs (step 1) to activate NF-κB or a non-NF-κB pathway to produce pro- IL-1β and pro-IL-18. Step 2 occurs when active caspase-1 cleaves pro-IL-1β and pro-IL-18 to produce mature cytokines IL-1β and IL-18. Mito-ROS also induce the formation of AGE, as a DAMP to activate NLRP3; mDNA also activates NLRP3 inflammasomes as a DAMP. Additionally, Mito-ROS trigger responses to hypoxia, activating HIF-1, and upregulate proinflammatory genes like TNF, IL-1β, IL-6, iNOS, MCP-1, and PAI-1. These cytokines activate the insulin signaling pathway and induce local and systemic insulin resistance (not shown). PDC is an important source of mitochondrial ROS, resulting in activation of NLRP3 inflammasomes as a DAMP. For simplicity and clarity, only part of signaling and cellular effects in macrophages are illustrated. AGE, advanced glycation end-products; ASC (apoptosis-associated speck-like protein containing a CARD), CI, ETC complex I; CII, ETC complex II; CIII, ETC complex III; CIV, ETC complex IV; CV, ETC complex V; ETC, electron transport chain; IL, interleukin; iNOS, inducible nitric oxide synthase; PM, plasma membrane; NF-κB, nuclear factor kappa-light-chain-enhancer of activated B cells; PAI-1, plasminogen activator inhibitor 1; PDC, pyruvate dehydrogenase complex; TNF, tumor necrosis factor; TLR, Toll-like receptor.

Excessive Mito-ROS drive inflammation through activation of NLRP3 inflammasomes (Figure 4) that can lead into insulin resistance and hypertension. Mito-ROS provide signaling to trigger NLRP3 inflammasome oligomerization or its relocation in proximity to the mitochondria (Misawa et al., 2013; Mastrocola et al., 2018). The NLRP3 inflammasome is a cytoplasmic multi-protein complex which is comprised of a sensor protein, an adaptor protein apoptosis-associated speck-like protein containing a caspase recruitment domain (ASC), and pro-caspase-1, a cysteine protease (Schroder and Tschopp, 2010). NLRP3 activation can be primed by toll-like receptors (TLRs) (step 1), which activates NF-κB or a non-NF-κB pathway to produce pro- IL-1β and pro-IL-18; step 2 involves the oligomerization of NLRP3 and recruitment of the adaptor protein ASC and pro-caspase-1 (Lee et al., 2020). Active caspase-1 or caspase-11 then cleaves pro-IL-1β and pro-IL-18 to produce mature cytokines IL-1β and IL-18 (Lee et al., 2020). IL-1β and IL-18 are among the most potent pro-inflammatory cytokines and are important in macrophage M1 (pro-inflammatory) polarization (Lee et al., 2020). Of note, increased ROS activate mediators of inflammation ultimately contributing to oxidative stress-induced metabolic diseases. There is increasing evidence that TLRs activate inflammation, which increases ROS production, leading to the vicious cycle of ROS and inflammation, and subsequent development and progression of chronic inflammatory diseases (Liang et al., 2013; Jha et al., 2018). Mito-ROS also induce the formation of AGEs, which function as a damage-associated molecular pattern (DAMP) or interact with its receptor, RAGE (receptor for advanced glycation end products) to activate NLRP3 (Misawa et al., 2013). The activation of NLRP3 contributes to the progression of several diseases, including MetS (Yu and Lee, 2016). Increased Mito-ROS production activates the NLRP3 inflammasome and the scavenging of Mito-ROS suppresses activation of the NLRP3 inflammasome (Yu and Lee, 2016). Pro-inflammatory cytokines, particularly IL-1β, act in an autocrine and paracrine manner to interfere with insulin signaling in adipose tissue, liver, skeletal muscle, and pancreas or induce β-cell dysfunction which leads to insulin deficiency and insulin resistance (Esser et al., 2014; Mastrocola et al., 2018). AGEs and other endogenous ligands interact with RAGE (Mastrocola et al., 2018), leading to the dysregulation of adipokines and cytokines, which results in insulin resistance and reinforces the already existing inflammatory responses (Gaens et al., 2014; Garay-Sevilla et al., 2021). In addition, Mito-ROS trigger responses to hypoxia, activating HIF-1 (Waypa et al., 2016), which induces inflammatory responses, via overexpression of inflammatory genes such as MCP-1, PAI-1, macrophage migration inhibition factor (MIF), TNF, IL-1β, IL-6, iNOS, and matrix metalloproteinases (Kim et al., 2014). These cytokines activate the insulin signaling pathway and local and systemic insulin resistance (Kim et al., 2014). Mito-ROS also suppress the expression of peroxisome proliferator-activated receptor gamma coactivator 1-α (PGC-1α), a co-activator of nuclear transcription factors, including nuclear respiratory factor (NRF)-1, PPARα, and PPARγ, that contribute to insulin resistance in MetS (Aroor et al., 2012). In mesenchymal stem cells of adipose tissue, ROS also activate transcription factor E2F, which activates PPARγ, stimulating adipocyte differentiation (Wang et al., 2015), indicating a role of Mito-ROS in regulating the cell cycle that induces adipogenesis and subsequently obesity (Rani et al., 2016). Mito-ROS also contribute to sustained vascular dysfunction and development of hypertension (Togliatto et al., 2017), which can be attenuated by inhibition of cyclophilin D, a regulatory subunit of the mitochondrial permeability transition pore, confirming a critical role of Mito-ROS in the development of hypertension (Itani et al., 2016).

Pyruvate dehydrogenase complex (PDC), an enzyme complex in mitochondria that converts pyruvate into acetyl-CoA, is an important source of ROS in the mitochondria (Holness and Sugden, 2003). PDC can be inactivated by phosphorylation with pyruvate dehydrogenase kinase (PDK)1-4 and activated by dephosphorylation with pyruvate dehydrogenase phosphatase (PDP)1–2 (Holness et al., 2003). In the brain, cardiac, liver, and skeletal muscle mitochondria, PDC produces ∼4 times more ROS than ETC Complex I (Quinlan et al., 2014; Brand, 2016). PDC activation in many cells increases aerobic respiration and ROS production (Yang et al., 2018; Huang et al., 2021). Patients with metabolic diseases have higher PDC activity. For example, PDC activity and protein expression in platelets are higher in diabetic patients than healthy volunteers (Michno et al., 2020).

Emerging evidence shows that sustained PDC activity induces chronic inflammation. In hepatic tissue, sterile inflammation leads to a 2.5–fold increase in the proportion of active, dephosphorylated form of PDC (Vary et al., 1986). PDC-induced inflammation occurs through activation of NLRP3 inflammasomes (Han et al., 2020), which, in turn, can be activated by increased ATP or ROS, as a DAMP, due to the increase in aerobic respiration induced by PDC (Yang et al., 2018; Huang et al., 2021). In mouse macrophages, inhibition of pyruvate flux decreases citrate, itaconate, and succinate levels and represses Tnf, iNos, Irg1, and Il1b gene expression (Meiser et al., 2016), indicating that PDC activation, indeed, promotes macrophage pro-inflammatory responses due to an increase in pyruvate influx. The chronic low-grade inflammation, in adipose tissue, liver, skeletal muscle, and vasculature of obese subjects, induces insulin resistance (Wu and Ballantyne, 2020). Mice lacking PDK2 and PDK4 have constitutively activated PDC, which increases glucose oxidation, reduces insulin-stimulated muscle glucose uptake, and decreases fatty acid oxidation. These result in increased re-esterification of acyl-CoA into diacylglycerol and triacylglycerol, with subsequent activation of PKC-θ and inhibition of insulin signaling and development of insulin resistance (Rahimi et al., 2014). However, a role of PDC in inflammation and insulin resistance is not accepted by everyone (Constantin-Teodosiu et al., 2015; Petersen et al., 2015), indicating the complicated nature of its role in the pathogenesis of MetS and warrants further investigation.

## Renal Dysfunction in Metabolic Syndrome

In addition to cardiovascular diseases and diabetes (Alberti et al., 2009; Nilsson et al., 2019), MetS also increases the risk of kidney injury and chronic kidney disease (Locatelli et al., 2006). The pathophysiologic mechanisms underlying MetS, such as oxidative stress, chronic inflammation, insulin resistance, hyperglycemia, dyslipidemia, and increased activity of the RAAS could all contribute to the renal dysfunction and pathogenesis of chronic kidney disease (CKD) (Zhang and Lerman, 2017; Lakkis and Weir, 2018), with oxidative stress playing a fundamental role (Ravarotto et al., 2018). CKD patients have oxidative stress, as shown by increased levels of oxidative stress markers, such as MDA and F2-isoprostanes, which further worsen the renal dysfunction (Tbahriti et al., 2013). The major sources of ROS in the kidney are also NADPH oxidases (Gill and Wilcox, 2006; Wan et al., 2016; Qaddumi and Jose, 2021) and mitochondria (Plotnikov et al., 2007; Lindblom et al., 2015).

NADPH oxidases, NOX1, NOX2, NOX4, and NOX5, are predominantly expressed in renal epithelial, tubulointerstitial, mesangial, and glomerular epithelial cells (Gill and Wilcox, 2006; Zeng et al., 2009; Qaddumi and Jose, 2021; Yang et al., 2021). Metabolic (hyperglycemia, dyslipidemia, ox-LDL, etc.) and non-metabolic stimuli (Ang II, aldosterone, vascular endothelial growth factor, etc.) activate the expression and activity of NADPH oxidases (Gill and Wilcox, 2006; Yang et al., 2020), resulting in ROS overproduction, which is involved in vascular, glomerular, renal tubular, and endothelial dysfunction, mesangial proliferation, and increase in renal sodium reabsorption, eventually resulting in hypertension and CKD (Wan et al., 2016; Ravarotto et al., 2018). A high glucose environment upregulates NOX4 expression, inactivates AMP-activated protein kinase, and promotes podocyte apoptosis via p53-and PUMA (p53 upregulated modulator of apoptosis)-dependent pathway (Eid et al., 2010), leading to podocyte injury, a critical early event of glomerulosclerosis. In high-fat diet fed mice, upregulation of NOX2, p47^phox^, and p67^phox^ expression induces hyperlipidemia-associated glomerular injury (Wan et al., 2016). Under MetS conditions, glomerular mesangial and vascular endothelial cells have decreased level of arginine or tetrahydrobiopterin, which promotes the electron transfer to oxygen, decreasing NO bioavailability, leading to endothelial dysfunction and mesangial expansion (Lee et al., 2013). NADPH oxidase activation, caused by Ang II and/or aldosterone, increases renal sodium transport, decreases NO bioavailability, and increases the proliferation of VSMCs and mesangial cells (Gill and Wilcox, 2006; Fazeli et al., 2012; Lee et al., 2013). Activation of NOX5 not only increases sodium transport in the renal proximal tubule (Yu et al., 2014; Yang et al., 2021) but also increases MCP-1 expression, macrophage infiltration, and secretion of proinflammatory chemokines and cytokines, and accelerates mesangial expansion and extracellular matrix protein accumulation, leading to glomerulosclerosis (Jha et al., 2017).

The genetic deletion or overexpression of NOX4, a major NADPH oxidase isoform in the kidney, does not affect the pathogenesis of CKD *in vivo* (Rajaram et al., 2019; Thallas-Bonke et al., 2020), indicating that other sources of ROS or additional yet unknown mechanism(s) are involved in the pathogenesis of renal dysfunction. The kidney utilizes a lot of energy with continuous oxidative phosphorylation within the mitochondria and as aforementioned, mitochondria and NADPH oxidases, are the major ROS sources in the cell (Murphy, 2009; Brand, 2016).

The abundance of mitochondria in the kidney produces the high energy demanded in the reabsorption and secretion of ions. However, an overly increase in Mito-ROS production causes mitochondrial dysfunction and mitochondrial damage that are involved in the pathogenesis of MetS (Zhang and Lerman, 2017; Podkowińska and Formanowicz, 2020). MetS affects renal mitochondrial structure and function through several different pathways (Zhang and Lerman, 2017; Podkowińska and Formanowicz, 2020). Metabolomics analysis demonstrated that the suppression of mitochondrial metabolism and activity in patients with MetS is associated with lower gene expression of PGC1α (a master regulator of mitochondrial biogenesis) and less mitochondria DNA and protein content in the kidney (Sharma et al., 2013). These findings are consistent with a recent observation that MetS patients with CKD (Jiang et al., 2019) have increased apoptosis and impaired ΔΨm in renal tubules but not podocytes (Jiang et al., 2019). Furthermore, oxidative stress-mediated perturbance of glycolysis and tricarboxylic acid cycle contributes to the tubular injury in MetS (Jiang et al., 2019). The injury in human renal proximal tubule cells caused by high-glucose in the medium is reversed by the mitochondria-targeted antioxidant, MitoQ (Xiao M. et al., 2017), that is related to the restoration of the Nrf2 expression, inhibition of the expression of Kelch-like ECH-associated protein (Keap1), and the interaction between Nrf2 and Keap1 (Xiao L et al., 2017). The high glucose-mediated injury of mouse renal mesangial cells can be attenuated by the tetrapeptide, SS-31, a mitochondria-targeted ROS scavenger (Hou et al., 2016). In these mouse mesangial cells exposed to high glucose, SS-31 decreased Mito-ROS generation, associated with a decrease in the expression of transforming growth factor β1, thioredoxin-interacting protein (TXNIP), BCL-2, apoptosis regulator (BAX), and cleaved caspase-3, and activation of p38 MAP kinase and cAMP-response element binding protein (CREB) (Hou et al., 2016).

Oxidative stress activates NLRP3 inflammasomes and quenching of Mito-ROS reverses NLRP3 activation (Yu and Lee, 2016). In MetS, the expression and activity of NLRP3 inflammasomes are increased in podocytes, glomerular endothelial cells, and tubular interstitial epithelial cells (Mastrocola, et al., 2018). Genetic deletion of NLRP3 ameliorates renal injury by preventing the early infiltration of immune cells, decreasing IL-1β and IL-18 expression and secretion, and proteinuria (Shahzad et al., 2015). NLRP3 inflammasomes are also involved in the disturbance of lipid metabolism in renal disease (Mastrocola, et al., 2018). NLRP3 activates sterol regulatory-element binding proteins (SREBPs) in lipotoxicity-driven inflammation and induces AGEs production. In Western diet-fed mice, the germline deletion of NLRP3 inactivates SREBPs, prevents renal lipid accumulation, and attenuates glomerular damage and proteinuria (Bakker et al., 2014). Hyperglycemia also promotes ROS through the enhanced glycolytic flux in mitochondria, induces AGEs production and TXNIP accumulation, which play a crucial role in NLRP3 inflammasome activation and increase in glomerular inflammatory injury (Tan et al., 2015).

Oxidative stress is also involved in the increased renal vascular resistance in the hypertension in MetS (Zeng et al., 2009; Ando and Fujita, 2012; Qaddumi and Jose, 2021). Induction of ROS generation in the renal medulla and cortex promotes hypertension (Cowley et al., 2015; Yang et al., 2020). Both NOX- and Mito-ROS, among others, contribute to the renal pathophysiology of hypertension (Zeng et al., 2009; Araujo and Wilcox, 2014; Loperena and Harrison, 2017; Yang et al., 2020; Yang et al., 2021). As mentioned previously, Ang II and aldosterone may be causal of the excessive ROS production in MetS. The chronic administration of Ang II type I receptor (AT_1_R) blocker decreases ROS production and vascular resistance (Gill and Wilcox, 2006; Daenen et al., 2019). Both blockade of AT_1_R and stimulation of dopamine receptors are reno-protective against oxidative stress by decreasing NADPH oxidase expression and activity (Cuevas et al., 2019; Daenen et al., 2019; Qaddumi and Jose, 2021; Yang et al., 2021). D_1_R activation increases the expression of the antioxidant, paraoxonase 2, in both lipid and non-lipid rafts in renal proximal tubule cells (Yang et al., 2015), D_2_R decreases ROS production through upregulation of paraoxonase 2 (Yang et al., 2012), DJ-1 (Cuevas et al., 2012), and another antioxidant, sestrin2 (Yang et al., 2014) in the kidney. In renal proximal tubule cells, D_5_R activation increases paraoxonase 2 (Yang et al., 2015), HO-1 (Lu et al., 2013), and Nrf2 (Jiang et al., 2018) protein expression. The involvement of Mito-ROS in the pathogenesis of hypertension is most likely caused by oxidative phosphorylation. Recently, Lee et al., in our laboratory, reported that PDC activity and expression are increased in both renal proximal tubule cells and cortical homogenates from spontaneously hypertensive rats compared with normotensive Wistar-Kyoto rats (Lee et al., 2014). This activation may be involved in the increase in the expression of sodium transporters and channels in nephron segments, including the renal proximal tubule (Knepper and Brooks, 2001; Wang et al., 2009; Maxwell et al., 2020; Pitzer et al., 2020).

## Reactive Oxygen Species-Targeted Therapeutic Implications

Because oxidative stress plays a fundamental role in the pathogenesis and progression of MetS, and the overproduction of ROS damages cellular macromolecules, there is increasing interest in developing therapeutic approaches targeting NADPH oxidases and mitochondria, or both to reduce ROS levels. Currently, the clinical approaches to reduce ROS in the treatment of MetS mainly involve changes in lifestyle, pharmacological drug therapy, and bariatric surgery (Murphy and Hartley, 2018; Bozi et al., 2020; Tun et al., 2020).

Decades of endeavors have targeted NADPH oxidases to lower ROS production (Araujo and Wilcox, 2014; DeVallance et al., 2019). The first generation of NADPH oxidase inhibitors are diphenyleneiodonium (DPI) and apocynin, which are not isoform-specific (Gill and Wilcox, 2006). Due to their broad profile of inhibition and side effects, more specific NADPH oxidase inhibitors have been developed, specifically GenKyoTex compounds, such as GKT137831 and GKT-136901 (Teixeira et al., 2017), triazolo pyrimidine compounds, such as VAS2870 and VAS3947 (Schramm et al., 2012), and the synthetic organoselenium ebselen, along with its congeners (DeVallance et al., 2019). Ebselen inhibits NOX1, NOX2, and NOX5, reduces vascular dysfunction, and improves insulin signaling in obese and diabetic rodents (Chen et al., 2009; DeVallance et al., 2019). NADPH oxidase isoform-specific inhibitors have been developed recently, specifically NOX1 inhibitors, such as ML171 and NoxA1ds, that block the interaction of NoxA1 with NOX1, reducing vascular resistance, improving endothelial function, and decreasing fat differentiation and migratory potential in MetS (Sela et al., 2015; Camargo et al., 2018). Nox2ds-tat, a peptide inhibitor of NOX2, interferes with p47phox docking to NOX2, causing the inhibition of ROS production (Csányi et al., 2011), reversal of vascular pathology, and restoration of insulin signaling (Sukumar et al., 2013; Quesada et al., 2015). Similar to the Nox2ds-tat, CPP11G and CPP11H, interfere with the translocation of p47phox to NOX2 in the plasma membrane, abolish ROS production, and attenuate endothelial cell inflammation and vascular dysfunction in an acute inflammatory mouse model (Li et al., 2019). All of these NADPH oxidase inhibitors are expected to be useful in the treatment of MetS.

Mitochondria-targeted antioxidants are reported to ameliorate MetS in experimental animals and humans (Bhatti et al., 2017; Murphy and Hartley, 2018; Bozi et al., 2020). Antioxidant compounds incorporated with ubiquinone or vitamin E, and the resulting compounds, MitoQ and MitoVit E, can specifically target the mitochondria to reduce oxidative injury and reverse mitochondrial dysfunction (Mao et al., 2011; Feillet-Coudray et al., 2014). The lipophilic triphenylphosphonium cation enables MitoQ to cross phospholipid bilayers, which leads to its accumulation within the mitochondria and reduction of mitochondrial ROS. MitoQ has been shown to reverse partially glucose intolerance, improve lipid metabolism, and restore mitochondrial activity in high-fat diet-fed Sprague-Dawley rats (Coudray et al., 2016), effects that were associated with a decrease in adipose tissue, and liver and body weights. In high-fat diet-fed mice, MitoVitE, another mitochondria-targeted antioxidant, protected the mitochondria against oxidative damage, improved subsarcolemmal mitochondrial density, and decreased systemic oxidative stress, manifested by an increase in plasma SOD activity and a decrease in urinary isoprostanes (Mao et al., 2011). Metformin, a drug widely used in the treatment of diabetes because of its ability to decrease the intestinal absorption of glucose and improve insulin sensitivity, decreases mitochondrial ROS, increases ADP:ATP ratio, induces AMPK activation, which then inhibits hepatic gluconeogenesis (Madiraju et al., 2018).

Antioxidants targeting both NADPH oxidases and mitochondria have been tested in clinical trials (Table 2). Although the role of ROS in the pathogenesis of MetS has been established in preclinical studies, the results in clinical studies have not been encouraging. Considering that high concentrations of ROS are harmful, reducing ROS levels should be beneficial. However, the degree of reduction of ROS to be beneficial is not known. The normal range more than likely varies in different cells at different times. Therefore, specific NADPH oxidase isoform or mitochondria-site antioxidants with cell- or tissue-specific drug delivery at a specific time is a promising therapeutic approach (Casas et al., 2020).

**TABLE 2:** Clinical trials of antioxidants in the therapy of metabolic syndrome or associated cardiovascular and chronic renal diseases.

Target	Antioxidant	NCT number	Subject recruitment status	Therapeutics	Main findings in clinical trials	Reference(s)
NADPH oxidases	Ebselen	NCT 00762671	Completed	Inhibitor	Anti-inflammatory	Beckman et al., (2016), Garland et al., (2020)
					No inhibition of ROS	
	GKT137831	NCT 02010242	Completed	Inhibitor	ND	NA
	Apocynin	NCT 03680638	Completed	Scavenger	ND	NA
		NCT 03680404	Completed		ND	NA
		NCT 03680573				
		NCT 04087655	Not yet recruiting		ND	NA
Both NADPH oxidases and Mitochondria	Tempol	NCT 03680638; NCT 03680404	Completed	Scavenger	ND	NA
Mitochondria	CoQ10	NCT 02407548	Completed	Inhibitor (cofactor of ETC)	CoQ10 increases TAC, reduces triglyceride, LDL-C, insulin resistance index, and blood pressure.	Zhang et al., (2018)
		IRCT2016011125949N1	Completed		CoQ10 decreases HOMA-IR, TC, LDL-C and increases HDL-C in diabetic patients.	Gholami et al., (2019)
		IRCT201502245623N35	Completed		CoQ10 has beneficial effects on serum insulin levels, HOMA-IR, HOMA-B and TAC in MetS patients.	Raygan et al., (2016)
		NCT 01412476	Completed		ND	NA
	Armolipid Plus (CoQ10)	NCT 01087632	Completed		Inhibition of Mito-ROS reduces blood glucose, cholesterol and triglycerides, and improves endothelial function and insulin resistance.	Solà et al., (2014) and Cicero et al., (2021)
		NCT 01562080				
	Armolipid Prev (CoQ10)	NCT 01293162	Completed		CoQ10 has anti-hypertensive, anti-dyslipidemic effects, reduces plasma homocysteine levels.	Izzo et al., (2010)
	MitoQ	NCT03586414	Suspended	Inhibitor	ND	NA
		NCT04334135	Recruiting		ND	NA
		NCT02364648	Recruiting		ND	NA

## Conclusion and Perspectives

MetS, which is a complex tangled web of oxidative stress with unhealthy states, including visceral obesity, hyperglycemia, dyslipidemia, and hypertension, occurs concomitantly in patients with elevated risk for cardiovascular diseases and chronic kidney diseases. As discussed in this review, the exact mechanisms underlying MetS are not clear. Oxidative stress, with inflammation, insulin resistance, and vascular endothelial damage, creates a pathophysiological condition that promotes the initiation and progression of MetS. Oxidative stress, the fundamental pathological basis for MetS, occurs because cellular anti-oxidative responses cannot counteract pro-oxidative effects. NADPH oxidases and mitochondria are the two major cellular sources for ROS production. ROS generated by NADPH oxidases may induce Mito-ROS generation, and vice versa, resulting in a vicious cycle. ROS, generated by NADPH oxidases and mitochondria, by themselves or by their interaction, in response to various exogenous and endogenous stimuli and metabolic alterations, trigger a series of adaptive and pathological responses, including the regulation of transcriptional factors and gene expression, and metabolic reprogramming. It is believed that low levels of ROS function as signaling molecules for physiological cellular functions whereas high levels of ROS are harmful to proteins, lipids, and nucleic acids. However, the boundary between the physiological signaling and pathological effects is unknown.

ROS, as DAMPs, activate NLRP3 inflammasomes, and therefore, mitochondria (and perhaps NADPH oxidases) can be considered as an integral component of the innate immune system to respond to intracellular and extracellular metabolic changes and stresses. It is assumed that ROS, generated from mitochondria, not only can cause the signaling and effects discussed above, but may also shape the metabolism and adaptive response of the immune system, including T and B cells. Further investigation is needed, not only on the mechanism underlying MetS but also the effects of cellular immunometabolism, to provide new paths for the therapeutic targeting for MetS.

Mito-ROS is usually linked to oxidative phosphorylation along the ETC. Recent evidence has demonstrated many sites that generate ROS in the mitochondria. For example, PDC produces more ROS than ETC Complex I. Consistent with the notion that oxidative stress is associated with the metabolic abnormality in MetS, renal proximal tubule cells from SHRs have higher PDC protein expression and activity than normotensive WKY rats; blood pressure also increases with the increase in PDC activity. PDC activity contributes to oxidative stress, but whether or not PDC activity increases sodium transport in the renal proximal tubule and other nephron segments warrants further investigation in patients and animals with MetS. The potential roles of PDC and the associated oxidative stress on inflammation, insulin resistance, alteration of glucose and lipid metabolism, adipocyte proliferation, endothelial dysfunction, and vascular resistance are largely unknown. The association of increased PDC activity with hyperglycemia, dyslipidemia, and even gut dysbiosis in the pathogenesis of MetS needs further investigation.

## Abbreviations

CoQ10, coenzyme Q10; ETC, electron transport chain; HOMA-B, homeostasis model of assessment β-cell function; HOMA-IR, homeostasis model of assessment insulin resistance; NA, not applicable; NCT, National Clinical Trial, ND, not determined; ROS, reactive oxygen species.
